# Mitochondria: More than Mitochondrial DNA in Cancer

**DOI:** 10.1371/journal.pmed.0030156

**Published:** 2006-03-28

**Authors:** Bora Baysal

**Affiliations:** **1**University of Pittsburgh School of MedicinePittsburgh, PennsylvaniaUnited States of America

In their *PLoS Medicine* article, entitled “A critical reassessment of the role of mitochondria in tumorigenesis,” Salas et al. [ 1] reviewed reports describing identification of mitochondrial DNA (mtDNA) mutations in several tumors. They identified many instances where the purported mutations in tumors corresponded to certain populational haplotypes, suggesting that contamination or sample mix-up could be a better explanation for these mtDNA variations found in tumors. This manuscript has important implications for this research field by questioning the validity of conclusions drawn in several high-profile publications that laid foundations for the role of mtDNA in cancer. While it is essential to investigate the origin of mtDNA variations found in certain tumors, the conclusion in the abstract that “the role of mitochondria in tumorigenesis remains unclarified” is simply incorrect.

The causal link between mitochondrial abnormalities and tumorigenesis was provided by the positional cloning of the hereditary paraganglioma gene at chromosome band 11q23 as the *SDHD* subunit gene of mitochondrial complex II (succinate dehydrogenase) in the year 2000 [ 2]. Since then, the role of mitochondria in cancer is further highlighted through identification of over 100 mutations in the *SDHB*, *SDHC*, and *SDHD* subunit genes in hundreds of index cases and families with hereditary and sporadic paragangliomas and pheochromocytomas [ 3]. Furthermore, fumarase gene mutations in a distinct hereditary tumor syndrome characterized by multiple skin and uterine leiomyomatosis and renal cell cancer—hereditary leiomyomatosis renal cancer (HLRCC)—further strengthened the role of mitochondria in cancer [ 4].

Although it is clear that Salas et al. question specifically the mutations in mtDNA of tumors, they did not acknowledge the causal link between mitochondria and cancer provided by the discovery of nuclear-encoded mitochondrial gene mutations. This is especially important because, in their unfortunate title and in their conclusion, the authors seem to make a sweeping statement against the role of mitochondria in cancer. It is essential to emphasize to readers that it is the mtDNA, but not mitochondria, which has a questionable role in tumorigenesis.
